# The role of *Mycobacterium tuberculosis* acetyltransferase and protein acetylation modifications in tuberculosis

**DOI:** 10.3389/fcimb.2023.1218583

**Published:** 2023-07-25

**Authors:** Yinxia Huang, Chuanzhi Zhu, Liping Pan, Zongde Zhang

**Affiliations:** Laboratory of Molecular Biology, Beijing Key Laboratory for Drug Resistant Tuberculosis Research, Beijing TB and Thoracic Tumor Research Institute, Beijing Chest Hospital, Capital Medical University, Beijing, China

**Keywords:** *Mycobacterium tuberculosis*, TB, post-translational modification, acetylation, N-acetyltransferase acetylation

## Abstract

Tuberculosis (TB) is a widespread infectious disease caused by *Mycobacterium tuberculosis* (*M. tb*), which has been a significant burden for a long time. Post-translational modifications (PTMs) are essential for protein function in both eukaryotic and prokaryotic cells. This review focuses on the contribution of protein acetylation to the function of *M. tb* and its infected macrophages. The acetylation of *M. tb* proteins plays a critical role in virulence, drug resistance, regulation of metabolism, and host anti-TB immune response. Similarly, the PTMs of host proteins induced by *M. tb* are crucial for the development, treatment, and prevention of diseases. Host protein acetylation induced by *M. tb* is significant in regulating host immunity against TB, which substantially affects the disease’s development. The review summarizes the functions and mechanisms of *M. tb* acetyltransferase in virulence and drug resistance. It also discusses the role and mechanism of *M. tb* in regulating host protein acetylation and immune response regulation. Furthermore, the current scenario of isoniazid usage in *M. tb* therapy treatment is examined. Overall, this review provides valuable information that can serve as a preliminary basis for studying pathogenic research, developing new drugs, exploring in-depth drug resistance mechanisms, and providing precise treatment for TB.

## 1 Introduction

In 2021, an estimated 10.6 million people (95% UI: 9.9–11 million) worldwide contracted tuberculosis (TB), marking an increase of 4.5% from 10.1 million (95% UI: 9.5–10.7 million) in 2020. During the same period, the TB incidence rate (new cases per 100,000 population per year) increased by 3.6% (WHO, 2022). TB is one of the most fatal infectious diseases, and its connection with HIV/AIDS is especially tragic (Riou and Althaus, 2020). HIV suppresses the immune system, making individuals more susceptible to *Mycobacterium tuberculosis* (*M. tb*) infections, hastening the progression to active TB, and increasing latent TB reactivation by 20-fold (Pawlowski et al., 2012; Wang Y., et al, 2022). It is concerning that HIV-infected individuals are more likely to develop drugresistant TB in Oceania and Eastern Europe. Additionally, HIVXDR-TB has become increasingly common among elderly people (Zhou et al., 2023).

Protein post-translational modifications (PTMs) are reversible mechanisms of cellular adaptation to changing environmental conditions. PTMs such as phosphorylation, acetylation, ubiquitination, and pupylation play a crucial role in mycobacterial virulence, pathogenesis, and metabolism. Approximately one third of the annotated *M. tb* proteome is modified post-translationally, and many of these proteins are essential for mycobacterial survival. Understanding the signaling pathways and PTMs may assist clinical strategies and drug development for *M. tb* (Budzik et al., 2020; Arora et al., 2021). Among PTMs, protein acetylation plays a crucial role in mycobacterial virulence, pathogenesis, and metabolism. In eukaryotes, protein acetylation is involved in almost all biological processes, including transcriptional regulation, protein translation, central metabolism, protein stability, signal transduction, and pathogen virulence (Carabetta and Cristea, 2017; Nakayasu et al., 2017; Christensen et al., 2019; Shvedunova and Akhtar, 2022). Recently, protein acylation has received increased attention due to its involvement in several mitochondrial, nuclear, and cytosolic processes (Glozak et al., 2005; Finkel et al., 2009; Norris et al., 2009; Kim and Yang, 2011). Protein acetylation is a dynamic equilibrium process in which the acetyl group of acetyl-coA is transferred to the N-a-amino group of protein or N-lysine protein group under the action of acetyltransferase or deacetylated transferase. Initially, it was believed to be an epigenetic modification of chromatin-related proteins, such as histones (Bernal et al., 2014). However, it is now suggested that acetylation modification plays important roles in biological processes. With the progress of mass spectrometry technology, the role of protein acetylation modification in the occurrence and development of diseases has become an important direction and focus of current research. Acetylation modification is a conserved post-translational modification discovered on histones in 1964 and is closely related to biological processes such as gene transcription regulation and protein function (Allfrey et al., 1964). In recent years, more and more studies have found that protein acetylation plays an indispensable role in the occurrence, development, and outcome of TB. Understanding the role and mechanism of new protein acetylation modification in the regulation of host anti-TB immunity is a current research focus on the epigenetic mechanism of TB. This may provide new targets for TB prevention, diagnosis, and host-directed therapy (HDT) for TB (Kilinç et al., 2021).

In this review, we will systematically discuss the new progress in the research of *M. tb* acetylation modification and related acetyltransferases. This will provide a theoretical basis and research ideas for exploring the development of novel anti-TB drugs targeting *M. tb* acetyltransferase, new mechanisms of drug resistance, and precise treatment. Additionally, we will explore how the metabolism of isoniazid (INH), a commonly used drug in TB therapy, depends on the N-acetyl transferase 2 (NAT2) enzyme.

In brief, the review will expatiate the following three parts:

1. acetylation modification of proteins & the role of acetyltransferase in *M. tb;*
2. acetylation modification of proteins in TB patients;
3. N-acetyltransferase acetylation (NAT) polymorphisms & TB treatment.

**Figure 1:**
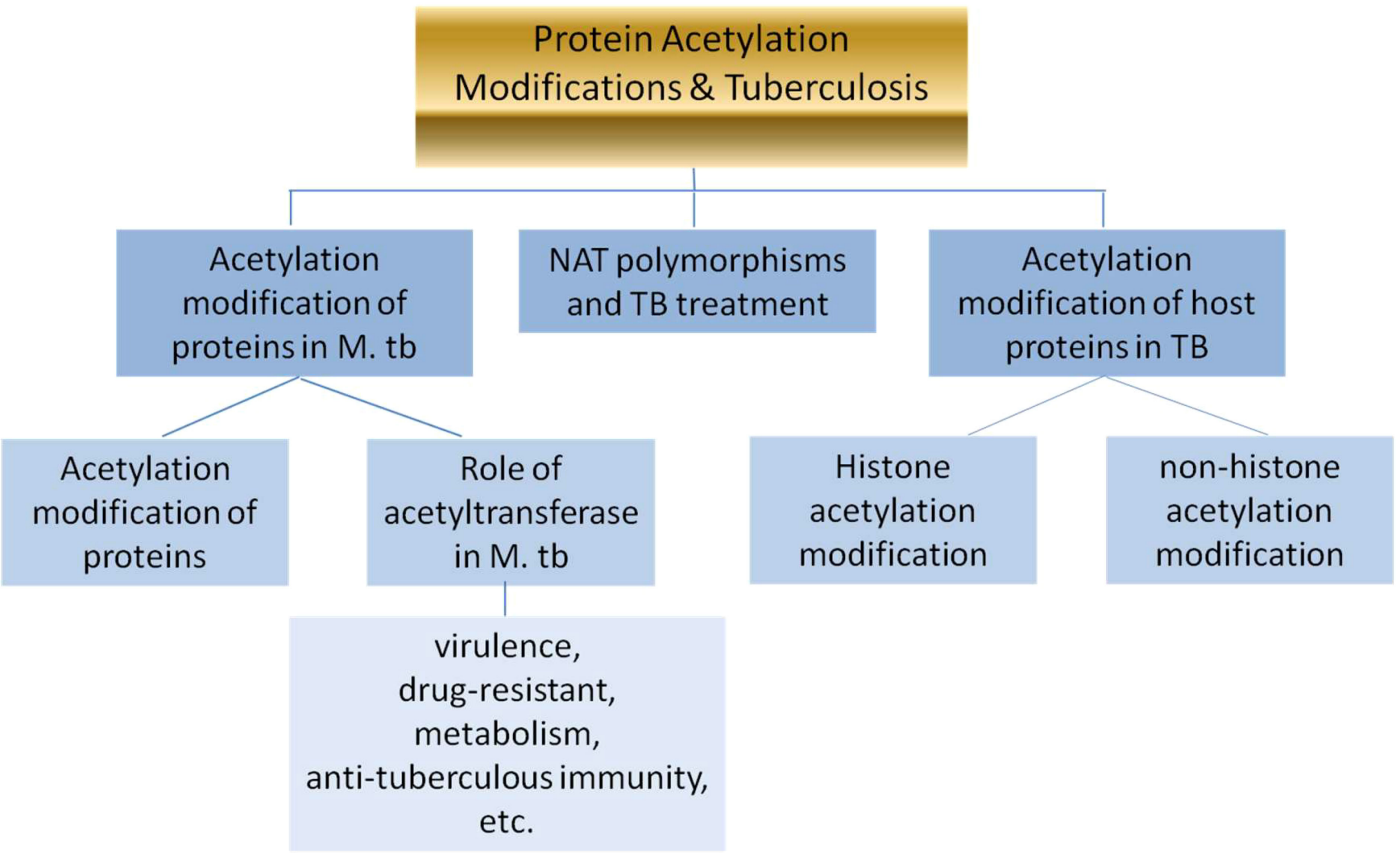
The whole flow diagram of the article.

The whole flow diagram of the article is below (**Figure 1**).

## 2 Acetylation modification of proteins in *M. tb*

### 2.1 Acetylated proteins and acetyltransferase in *M. tb*

Acetylation modification of proteins is a critical mechanism of cellular adaptation to changing environmental conditions, and is also implicated in the virulence, pathogenesis and metabolism of M. tb. The recent study has identified 1128 acetylation sites on 658 *M. tb* proteins, and Gene Ontology (GO) analysis of the acetylome revealed that acetylated proteins are involved in the regulation of diverse cellular processes including metabolism and protein synthesis (Xie et al., 2015). Singh KK. et al. showed that acetylation of response regulator protein MtrA inhibited phosphorylation modifications thereby promoting division of *M. tb* (Singh et al., 2020). The acetyltransferase of *M. tb* is involved in the modification of various small molecular substrates, including antibiotics, amino acids, and other molecules, thereby regulating transcription, translation, protein folding, and metabolic pathways. The bioinformatics analysis revealed the existence of 47 potential acetyltransferases in *M. tb* (**Table 1**), among which three genes, namely Rv2747, Rv3341, and Rv1653, encode essential acetyltransferases (Xie et al., 2019). Additionally, *M. tb* acetyltransferase can interact with host immune signaling proteins and modulate the host innate immune response against TB (Burckhardt and Escalante-Semerena, 2020. Shvedunova and Akhtar, 2022). The identification of acetylated proteins and acetyltransferases in *M. tb* provides a theoretical basis and research ideas for the development of novel anti-TB drugs targeting *M. tb* acetyltransferase, new mechanisms of drug resistance, and precise treatment.

**Table 1:** 47 potential acetyltransferases of *M. tb*.

Gene	Protein	Substrate	Function	Reference
Rv0032 Rv0133 Rv0243 Rv0262c Rv0408	bioF2 Rv0133 FadA2 Aac Pta	unknown unknown acetyl-CoA unknown acetate	involved in biotin biosynthesis (at the first step) acetylation lipid metabolism, virulence related gene involved in lipid degradation confers resistance to aminoglycosides conversate acetate to acetyl-CoA	Minato et al. (2019) DeJesus et al. (2017) Mattow et al. (2003) DeJesus et al. (2017) Minato et al. (2019)
Rv0428c Rv0730	Rv0428c Rv0730	unknown unknown	acetylation, regulation of bacterial survival under stress acetylation	Sharma et al. (2022) Målen et al. (2010)
Rv0802c	Rv0802c	unknown	succinylation and acetylation of nucleus-associated proteins	Anand et al. (2021); Vetting et al. (2008)
Rv0819	MshD	unknown	mycothiol biosynthesis, serological diagnostic marker	Zeitoun et al. (2017)
Rv0859 Rv0914c	FadA Rv0914c	acetyl-CoA unknown	involvement in lipid degradation, inhibiting host fatty acid metabolism and anti-tuberculosis immune response under hypoxia conditions involvement in degradative pathways such as fatty acid BETA_OXIDATION	Yang et al. (2021); Minato et al. (2019)
Rv0919 Rv0995	TacT RimJ	tRNA ribosomal protein S5	acetylate tRNA acetylation	Tomasi et al. (2022) Griffin et al. (2011)
Rv0998	Rv0998	lysine	regulate M.tb metabolism to adapt to anoxic environment	Yang et al. (2018)
Rv1018c	GlmU	UDP-n-acetylglucosamine pyrophosphorylase	IL-8 binding effector protein, promote M. tb invasion of human neutrophils	Dziadek et al. (2016)
Rv1074c Rv1135A Rv1323	FadA3 Rv1135A FadA4	unknown unknown unknown	lipid metabolism involved in lipid degradation involvement in lipid degradation	Minato et al. (2019) Minato et al. (2019) DeJesus et al. (2017)
Rv1347c Rv1505c Rv1565c	MbtK Rv1505c Rv1565c	lysine unknown unknown	regulates acylation of mycobacterin unknown unknown	Madigan et al. (2015) Minato et al. (2019) Minato et al. (2019)
Rv1653 Rv1867	ArgJ Rv1867	ornithine unknown	catalyze arginine biosynthesis involvement in lipid degradation	Sankaranarayanan et al. (2009) Minato et al. (2019)
Rv2170	Rv2170	aminoglycoside antibiotics	acetylation, carbon source and energy metabolism regulation, isoniazid acetylation modification	Lee et al. (2017); Arun et al. (2020)
Rv2215	DlaT	dihydrothioctylamine	virulence factor, metabolism and nitrosation stress regulation	Shi and Ehrt (2006)
Rv2335	CysE	serine	regulate the growth rate of mycobacterium	Qiu et al. (2014)
Rv2416c Rv2669 Rv2704	Eis Rv2669 Rv2704	aminoglycoside antibiotics unknown unknown	regulate host protein acetylation modification and immune response, acetylate aminoglycosides mediated drug resistance acetylation unknown	Tamman et al. (2014); Logesh et al. (2022) Minato et al. (2019) Minato et al. (2019)
Rv2747 Rv2775 Rv2851c	ArgA Rv2775 Rv2851c	glutamic acid unknown unknown	catalyze L-arginine biosynthesis acetylation acetylation	Yang et al. (2017); Das et al. (2019) Minato et al. (2019) Minato et al. (2019)
Rv2867c Rv3027c Rv3034c	Rv2867c Rv3027c Rv3034c	unknown unknown unknown	acetylation acetylation regulate macrophage oxidative stress response	Betts et al. (2002) DeJesus et al. (2017) Ganguli et al. (2020); Behera et al. (2022)
Rv3216 Rv3225c	Rv3216 Rv3225c	unknown unknown	acetylation acetylation, phosphorylation,	Griffin et al. (2011) Draker et al. (2003)
Rv3338	Rv3338	unknown	unknown	Minato et al. (2019)
Rv3341	MetA	homoserine	involved in the biosynthesis of methionine, catalyzes acylation of L-homoserine, substrate dependent transferase and hydrolase activity	Maurya et al. (2020)
Rv3420c	RimI	alanine ribosomal protein	Acetylates the N-terminal alanine of ribosomal protein S18	Pathak et al. (2016)
Rv3423.1	unknown	histidine	regulate the K9/K14 acetylation modification of host histone H3, manipulates the expression of host genes involved in anti-inflammatory responses	Jose et al. (2016)
Rv3523 Rv3525c	Ltp3 Rv3523c	unknown unknown	probably involved in lipid metabolism probably involved in lipid metabolism unknown	Van der Geize et al. (2007) Kelkar et al. (2011)
Rv3546 Rv3556c Rv3566c	FadA5 FadA6 Nat	acetyl-CoA unknown arylamine	virulence factor, regulate cholesterol side chain catabolism involved in lipid degradation involved in lipid degradation	Could have a role in acetylating, and hence inactivating, the antitubercular drug isoniazid	Lu et al. (2017); Jaiswal et al. (2018) de Souza et al. (2011) DeJesus et al. (2017) Kelkar et al. (2011)
Rv3700c	EgtE	unknown	probably involved in cellular metabolism	Sassetti et al. (2003); Saini et al. (2016)

### 2.2 Acetyltransferase associated with *M. tb* virulence

The success of *M. tb* as a pathogen is partly attributed to its ability to sense and respond to dynamic host microenvironments. Protein acetylation modification plays a key role in bacterial virulence and pathogenicity (Ren et al., 2017). Various *M. tb* acetyltransferases have been identified and confirmed to act as virulence factors. The acetyltransferase Pat, encoded by Rv0998, has been shown to have acetylase activity that is directly regulated by cAMP binding *in vitro* (Nambi et al., 2010; Xu et al., 2011). Studies have demonstrated that the acetylation of a conserved lysine 193 (K193) within the C-terminal DNA-binding domain of the cyclic AMP (cAMP) receptor protein (CRP) reduces its DNA-binding ability and inhibits transcriptional activity. The reversible acetylation status of CRP K193 has been shown to significantly affect mycobacterial growth phenotype, alter the stress response, and regulate the expression of biologically relevant genes (Di et al., 2023). Shi SP. et al. (Shi and Ehrt, 2006) generated a Rv2215/*dlaT* knockout strain and tested its ability to grow, resist nitrosative stress, and cause disease in mice, which demonstrated that Rv2215/dlaT is required for optimal growth of *M. tb*. *DlaT* encodes dihydrolipoamide acyltransferase, which together with the pyruvate dehydrogenase E1 component (AceE) and dihydrolipoamide dehydrogenase (Lpd) constitutes pyruvate dehydrogenase (PDH) in *M. tb.* PDH catalyzes the oxidation of pyruvate by NAD to acetyl-coenzyme A (acetyl-CoA) and CO2. Acetyl-CoA then feeds into the tricarboxylic acid (TCA) cycle.

Although the existence of *M. tb* acetyltransferases as virulence factors has been discovered, the specific targets of these acetyltransferases and the exact molecular mechanisms affecting *M. tb* virulence remain to be studied and clarified.

### 2.3 Acetyltransferase associated with drug-resistant TB

The continuing spread of drug-resistant TB is one of the most urgent and difficult challenges facing global TB control. Studies have found that the activity of most of the existing ten kinds of anti-TB drugs, such as aminoglycosides, chloramphenicol, streptomycin, fluoroquinolones and other drugs is regulated by acetylation modification (Schwarz et al., 2004; Reygaert, 2018). For instance, in addition to mutations in katG, nhA, ahpC, kasA, and ndh genes, isoniazid (INH) resistance is associated with acetyltransferase Rv2170, which catalyzes the transfer of acetyl group from acetyl CoA to INH to form acetylated INH (Silva et al., 2003). The acetylated INH is then decomposed into isonicotinic acid and acetylhydrazine, overcoming INH toxicity and producing resistance (Arun et al., 2020). Furthermore, acetylation modification can also affect the metabolic rate of INH *in vivo*, thereby affecting its therapeutic effect in different individuals (Jing et al., 2020).

The enhanced intracellular survival (Eis) protein encoded by *M. tb* is an acetyl transferase that targets aminoglycoside antibiotics. Zaunbrecher et al. and Houghton et al. have found that EIS-mediated acetylation modification can inactivate kanamycin, capreomycin and other drugs (Zaunbrecher et al., 2009; Houghton et al., 2013). Reeves et al. found that transcription regulator WhiB7 promoted kanamycin acetylation by enhancing the transcription of Eis genes, and Eis itself was also regulated by acetylation modification (Reeves et al., 2013). Moreover, small molecule inhibitors targeting Eis have also been developed rapidly in recent years. Garzan et al. found that Eis inhibitors can be effectively applied in kanamycin adjuvant combination therapy, which provides a new solution for drug resistance (Garzan et al., 2016; Garzan et al., 2017; Punetha et al., 2020). However, the effect of Eis acetylation on its own activity and its mechanism in regulating aminoglycoside drug resistance remains unclear (Birhanu et al., 2017). Additionally, it has been reported that Rv0262c encoded aminoglycoside 2’-n-acetyltransferase can also acetylate all known aminoglycoside antibiotics, including ribomycin, neomycin B, gentamicin and tobramycin containing 2’ amino, etc. (Hegde et al., 2001). Correspondingly, Rv3225c-encoded acetyltransferase has a low level of aminoglycoside modification activity on aminoglycoside antibiotics, which can lead to resistance of *M. tb* to aminoglycoside antibiotics through acetylation modification (Kim et al., 2006). Meanwhile, N-acetyl cysteine can artificially increase respiration and additional ROS accumulation, which enhances moxifloxacin lethality in *M. tb*-infected cultured macrophages and mice. Addition of ROS stimulators to fluoroquinolone treatment of TB constitutes a new direction for suppressing the transition of MDR-TB to XDR-TB (Singh et al., 2022).

In summary, the regulation of drug acetylation modification by *M. tb* through acetyltransferase is an important cause of drug resistance, according to the studies mentioned above. These studies suggest that small molecule inhibitors targeting *M. tb* acetyltransferase activity can be developed directly as new anti-TB drugs and can also promote the anti-TB effect of existing drugs by enhancing their sensitivity or preventing drug tolerance.

### 2.4 Acetyltransferase associated with *M. tb* metabolism

In the metabolic pathway, approximately 90% of metabolic enzymes in the metabolic pathway, including tricarboxylic acid cycle, gluconeogenesis, glycolysis, glycogen metabolism, fatty acid metabolism, and urea cycle, undergo acetylation modification (Zhao et al., 2010). Rv2170 has been found to possess lysine acetyltransferase activity, which can affect the glyoxylic acid metabolism or tricarboxylic acid cycle by reducing the lysine residues of Isocitrate lyase or Isocitrate dehydrogenase through acetylation modification (Lee et al., 2017). Moreover, the deacetylation of DosR at K182 promotes the hypoxia response in *M. tb* and enhances the transcription of DosR-targeted genes. Rv0998 has been identified as an acetyltransferase that mediates the acetylation of DosR at K182. Deletion of Rv0998 also promoted the adaptation of *M. tb* to hypoxia and the transcription of DosR-targeted genes. Mice infected with an *M. tb* strain containing acetylation-defective DosR^K182R^ had much lower bacterial counts and less severe histopathological impairments compared with those infected with the wild-type strain (Yang et al., 2018). Additionally, Rv0998 has been shown to regulate carbon flux, change oxidation reaction, and reduce tricarboxylic acid cycle reaction, which may contribute to *M. tb* survival in mice (Bi et al., 2018; Rittershaus et al., 2018). The acetylase activity of Rv0998 is regulated by metabolism-related products, including cAMP, acetyl-CoA, and the deacetylase Rv1151c (Bi et al., 2018). These findings suggest that targeting *M. tb* acetyltransferase in its own metabolic pathway could be a potential pathway for anti-TB therapy.

Furthermore, TB is linked to human metabolism, and individuals with diabetes and other metabolic disorders have a higher risk of *M. tb* infection (Bernal et al., 2014). Therefore, investigating the effect of *M. tb* acetyltransferase on host metabolism could be a promising new strategy for developing anti-TB therapy targeting metabolism-related enzymes.

### 2.5 Acetyltransferase associated with host anti-tuberculous immunity

TB arises from the interplay between bacterial virulence and host immunity. The virulence factors of *M. tb* enable it to evade the host immune system and survive within the host (Zhu et al., 2019). For example, Kim et al. (Kim et al., 2012) found that Eis protein inhibits JNK-dependent autophagy, phagosome maturation and Reactive Oxygen Species (ROS) production through acetylation of DUSP16/MKP-7 at K55 site. Duan Liang et al. (Duan et al., 2016) found that Eis protein inhibits macrophage autophagy by increasing histone H3 acetylation, up-regulating IL-10 expression, and then activating AKT/mTOR/P70S6K pathway. Rv3423.1, a novel histone acetyltransferase from *M. tb*, has been shown to mediate acetylation at the H3K9/K14 positions by co-localizing with the host chromatin in the nucleus. By binding to the host chromatin, Rv3423.1 may manipulate the expression of host genes involved in anti-inflammatory responses, allowing *M. tb* to evade clearance and survive in the intracellular environment (Jose et al., 2016). Another protein secreted by mycobacteria under hypoxia, FadA (Fatty-acid degradation A), acts as an acetyltransferase that converts host acetyl-CoA to acetoacetyl-CoA. This reduces the acetyl-CoA level and suppresses H3K9Ac-mediated expression of the host proinflammatory cytokine Il-6, thereby promoting granuloma progression (Yang et al., 2021). Eis also acetylates *M. tb* HU (MtHU), which leads to reduced DNA interactions and altered DNA compaction capacity of NAP (Ghosh et al., 2016). Overexpression of Eis can result in excessive acetylation of HU and genomic decompression. Given the importance of HU for *M. tb* survival, it is possible that its acetylation by Eis is also linked to drug resistance and survival.

Thus, understanding the role of acetyltransferases in host immunity against TB may offer a new therapeutic approach to TB infection.

## 3 Acetylation modification of host proteins in TB

### 3.1 Protein acetylation modification and diseases

Protein acetylation and deacetylation is catalyzed by protein acetyltransferases and deacetylases, respectively, of which several families exist. There are two types of protein acetylation: the acetylation of proteins at the ε-amino group and the acetylation of the a-amino group of the N-terminal amino acid (Bernal et al., 2014; Khadela et al., 2022). While the acetylation of the α-amino group of the N-terminal amino acid of proteins is very rare in bacteria, it is frequent in eukaryotes (30 - 80% of proteins) and archaea (14 - 29% of proteins) (Polevoda and Sherman, 2003; Soppa, 2010). On the other hand, the acetylation of proteins at the ε-amino group of internal lysine residues is a widely distributed PTMs, frequent in all domains of life. In eukaryotes, the physiological relevance of N-ε-lysine protein acetylation is well demonstrated. It has been demonstrated that the 8-amino group of multiple lysine side chains in histones can be acetylated to manipulate gene expression by regulating chromatin tightness or influencing transcription factor binding in promoters and distal enhancers, as well as histone DNA interactions (Roth et al., 2001; Yuan et al., 2009; Bannister and Kouzarides, 2011; Barnes et al., 2019). For the first time, Choudhary et al. identified the existence of acetylation modification at 3600 lysine sites on 1750 proteins, suggesting that lysine acetylation has a wide range of regulatory effects (Choudhary et al., 2009). Non-histone acetylation plays a key role in physiological and pathological processes, including the regulation of enzyme activity, protein degradation, protein interaction, subcellular localization, chromatin regulation and metabolism (Drazic et al., 2016; Narita et al., 2019). Abnormal protein acetylation or deacetylation is closely related to many diseases, such as leukemia, cancer, diabetes, infectious diseases, cardiovascular and nervous system related diseases and so on (Timmermann et al., 2001; Morales-Tarré et al., 2021).

### 3.2 Histone acetylation modification induced by *M. tb* infection

During *M. tb* infection, the host anti-TB immune response was regulated by *M. tb*-induced gene expression, which is one of the strategies for its intracellular survival and progression of TB. Research reports that *M. tb*-infected macrophages inhibit histone H3 acetylation (H3Ac) in the interleukin-12B (IL-12B) promoter region, leading to down-regulation of IL-12B expression and inhibition of Th1 type immune response. This promotes *M. tb* survival in the host (Chandran et al., 2015). Wang et al. found that *M. tb* infection inhibit HLA-DR gene expression by regulating the recruitment of HDAC complex in the HLA-DR promoter to enable its intracellular survival (Wang et al., 2005). Chen et al. found that the expression of H3K14Ac in peripheral blood lymphocytes of TB patients was reduced, especially the specific enrichment in the promoter region of TNF-α and IL-12B was decreased, which was related to the survival rate of TB patients (Chen et al., 2017). In addition, the up-regulated expression of HDAC1 inhibits the expression of H3K14Ac and plays a role in the outcome of active pulmonary TB and its clinical treatment. Moores et al. found that *M. tb* regulates the expression of matrix metalloproteinases (MMP-1 and MMP-3) via HDAC and histone acetyltransferase (HAT) activity and the manipulation of histone acetylation modification, which is a key factor in TB immune pathogenesis (Moores et al., 2017). These studies suggest that acetylation of histones, or acetylation of specific lysine sites, is associated with intracellular survival of *M. tb* and the development of TB.

Recently, the studies of histone acetylome-wide associations (HAWAS) showed that there were at least 2000 differences in acetylation sites associated with differential gene expression in the whole genome of peripheral granulocytes and monocytes of TB patients and healthy people. Histone acetylation quantitative trait locus (haQTL) analysis revealed candidate causal immunophenotypic changes in different populations of granulocyte and monocyte haQTL. *M. tb* infection regulates the differential enrichment of the inward rectifier potassium channel subfamily promoter J member 15 (CNJ15) of H3K27Ac, which enhances cell apoptosis and promotes *M. tb* clearance *in vitro* (Del Rosario et al., 2022). On the other hand, trained immunity, proposed by Netea et al. and Joosten et al., has become an important new evaluation index system for host immune protection induced by TB vaccines (Netea et al., 2016; Joosten et al., 2018). Post-immunization mediated trained immunity (mainly affecting H3K27Ac) of BCG or MTBVAC, an active *M. tb* candidate vaccine, can enhance the production of cytokines by monocytes and thus provide immune protection (Tarancón et al., 2020; Sheng and Cristea, 2021). Li et al. found that BCG infection can up-regulate the expression level of p300 in mature THP-1 cell lines and regulate the acetylation level of histone H3 and AP-2α. It was further demonstrated that trichostatin A (TSA), a broad-spectrum histone deacetylase inhibitor, enhances the enrichment of the toll-like receptor2 (TLR2) promoter by regulating the acetylation of AP-2α. Furthermore, promoter transcriptional activity was increased to up-regulate TLR2 gene expression (Li et al., 2013). Pennini et al. found that activation of TLR2 inhibits IFN-induced acetylation of histones H3 and H4 (Pennini et al., 2006). Therefore, targeted regulation of acetylation of specific lysine sites in histones may be an important way to enhance the host’s effective resistance to *M. tb* infection and/or promote immune clearance.

### 3.3 *M. tb* infection induced host non-histone acetylation modification

In addition to histone acetylation, non-histone acetylation also plays a crucial role in regulating cellular processes. Like histone proteins, non-histone proteins are also modified by histone acetyltransferases and HDACs. Various studies have reported that non-histone acetylation plays an essential role in the occurrence, development, and outcome of infectious diseases caused by viruses, bacteria, and other pathogens such as DNA virus, influenza virus, rabies virus, and *Salmonella* typhi infection (Wu et al., 2010; Green et al., 2018; Song et al., 2020). Furthermore, non-histone acetylation modification has been found to regulate autophagy, apoptosis, and inflammasome activation in innate immune responses (Wan et al., 2017; Son et al., 2020; Pagán et al., 2022) Autophagy-related proteins such as ATG5, ATG7, ATC8, and ATG12 can be acetylated by p300, leading to inhibition of autophagy (Lee and Finkel, 2009; Battaglioni et al., 2022; Wang D. et al., 2022). On the other hand, NAD^+^-dependent histone deacetylase Sirt1 can deacetylate ATG5, ATG7, and LC3, promoting autophagy occurrence (Lee et al., 2008).

In the process of *M. tb* infection, studies have shown that Sirt1 activation induced by *M. tb* infection can activate autophagy by directly mediating MAP1LC3B/LC3B deacetylation, which may limit the growth of intracellular *M. tb* (Iqbal et al., 2021). These findings suggest that acetylated autophagy-related proteins play a key role in regulating autophagy activation and inhibition, and the role of autophagy in host anti-*M. tb* infection has been established (Pellegrini et al., 2021). Non-histone deacetylation mediated by Sirt family proteins is suggested to be significant in understanding *M. tb*-mediated inflammatory response and discovering new drug targets (Cheng et al., 2017; Bhaskar et al., 2020; Yang et al., 2021; Yang et al., 2022). In another recent experiment, Brandenburg J. et al. showed that Wnt family member 6 (WNT6) promotes foam cell formation during TB by regulating key lipid metabolism genes including Acetyl Coenzyme A Carboxylase (ACC2). These findings open new perspectives for host-directed adjunctive treatment of pulmonary TB (Brandenburg et al., 2021).

Based on the above studies, non-histone acetylation modification is shown to be effective in host anti-TB immunity. Additionally, non-histone modifications involved in cell signal transduction, protein interaction, protein aggregation, protein degradation, and subcellular localization may also play a critical role in regulating host anti-TB immunity during *M. tb* infection.

## 4 N-acetyltransferase acetylation polymorphisms and TB treatment

### 4.1 Introduction of N-acetyltransferase acetylation

Arylamine N-acetyltransferase comprises N-acetyltransferase 1 (NAT1) and N-acetyltransferase 2 (NAT2) in humans (Hein et al., 2022). NAT2 is mainly expressed in the liver and the GI tract (Husain et al., 2007), and is responsible for the N-acetylation polymorphism observed in human populations (Weber and Hein, 1985; McDonagh et al., 2014; Agundez and Garcia-Martin, 2018; Mitchell, 2020). There are several single nucleotide polymorphisms (SNPs) in the coding exon of the NAT2 gene, which are inherited as NAT2 haplotypes and genotypes and confer rapid, intermediate, and slow acetylator phenotypes that modify drug metabolism (Hein, 2009; Hein and Millner, 2021).

### 4.2 Relationship between NAT2 genotype with drug metabolism and toxicology

Although isoniazid (INH) remains one of the major first-line drugs, the extensive use of INH to treat active and latent TB infections is compromised by INH-induced hepatotoxicity and liver failure (Hall et al., 2009; Sterling et al., 2020). The NAT2 genotype dependent pharmacokinetic parameters measured in human subjects have been confirmed by measurement of INH N-acetylation both *in vitro* and *in situ* in cryopreserved human hepatocytes and the TB patient (Doll et al., 2017; Hein and Millner, 2021). A study conducted in Indonesia revealed that patients with TB and the slow-acetylator phenotype caused by NAT2 variants are highly susceptible to drug-induced liver injury caused by anti-TB drugs, confirming the association between slow-acetylator NAT2 variants and susceptibility to drug-induced liver injury in an Indonesian population (Yuliwulandari et al., 2016). Furthermore, recent studies concluded that INH N-acetylation in human subjects differs significantly with respect to rapid, intermediate, and slow acetylator NAT2 genotypes in terms of plasma half-life, bioavailability (area under the curve), plasma metabolic ratio of INH to N-acetyl-INH, and clearance. The meta-analysis studies report that slow acetylators were significantly more likely to experience hepatotoxicity from INH treatment for TB than rapid acetylators (Khan et al., 2019; Richardson et al., 2019).

### 4.3 NAT2 polymorphisms and guide isoniazid dosing for TB treatment

Personalized therapy, also known as host-directed therapy (HDT), is being developed in many recent studies for conditions such as TB caused by *M. tb*. Epigenetic processes, including acetylation modification, play a crucial role in the development of personalized HDT (Marimani et al., 2018). One of the most focused themes among these studies is NAT2 polymorphisms. The paradigm for NAT2 phenotype-dependent dosing strategies is presented as a value of pharmacogenomics-guided isoniazid therapy for the prevention and treatment of TB. Béranger Agathe et al. have demonstrated that NAT2 genotype is the most impactful factor of INH metabolism, compared with low-birth-weight (LBW) and preterm infant born (Béranger et al., 2022). Phenotype-dependent dosing strategies aim to reduce the risk of adverse reactions, increase therapeutic efficacy, reduce costs, and improve patient care and disease prevention. Several studies have proposed pharmacogenomics-guided INH therapy for TB (Matsumoto et al., 2014; Jung et al., 2015; Choi et al., 2017; Motta et al., 2018; Suvichapanich et al., 2018; Jing et al., 2020). A recent study conducted in the USA and Brazil found that the clearance rates of INH were lowest in predicted slow acetylators (median 19.3 L/hr), moderate in intermediate acetylators (median 41.0 L/hr), and highest in fast acetylators (median 46.7 L/hr) (Verma et al., 2021). Moreover, there are significant differences in the distribution of NAT2 gene polymorphisms among different nationalities and races, the anti-TB treatment regimens adopted by patients are different, and the tolerance and exclusion standards of the subjects are different (Suarez-Kurtz et al., 2016; Zahra et al., 2020; Zhang et al., 2021). Depending on the NAT2 genotype of the patients, several studies have evaluated isoniazid doses of 2.5 mg/kg (0.5 times standard dose), 5 mg/kg (standard dose), and 7.5 mg/kg (1.5 times standard dose) for slow, intermediate, and fast metabolizers, respectively. As a result, a better treatment success rate was achieved, and the occurrence of liver function injury was reduced (Azuma et al., 2013; Huerta-García et al., 2020).

All these results suggest that understanding the diversity of drug-related genetic markers is critical for individualized drug-gene therapy programs in ethnic minorities in China and populations highly mixed with these ethnic groups. The above studies could make personalized TB treatment dosing available in reality. Pharmacogenomic-guided dosing can help achieve consistent drug levels and improve clinical outcomes.

## 5 Conclusions and future directions

As one main type of epigenetics in TB, acetylation plays a crucial role in aiding *M. tb* survival in the host, rendering the host vulnerable to the pathogen, and activating the host’s immune system against the invading pathogen. Hence, the study of acetylation processes is crucial for comprehending the progression of *M. tb*, identifying ideal candidates for therapeutic targets, minimizing drug toxicity, and monitoring the efficacy of administered therapy in developing personalized medication regimens. There are still some limitations of current research on protein acetylation within TB. We still lack dynamic change analysis of the acetylated protein expression. Future research may focus on the dynamic changes of acetylated protein expression in *M. tb* and host at different time points, which will provide more effective HDT targets for drug treatment of TB.

## Author contributions

YH and CZ contributed to conception, design and drafting the manuscript. LP and ZZ contributed to conception and critically revised the manuscript. All authors contributed to the article and approved the submitted version.
